# Neuronal regulation of bone marrow stem cell niches

**DOI:** 10.12688/f1000research.22554.1

**Published:** 2020-06-16

**Authors:** Claire Fielding, Simón Méndez-Ferrer

**Affiliations:** 1 Haematology, University of Cambridge, Cambridge, UK; 2 Wellcome-MRC Cambridge Stem Cell Institute, Cambridge, UK; 3 National Health Service Blood and Transplant, Cambridge, UK

**Keywords:** hematopoietic stem cell, niche, autonomic nervous system, adrenergic, cholinergic

## Abstract

The bone marrow (BM) is the primary site of postnatal hematopoiesis and hematopoietic stem cell (HSC) maintenance. The BM HSC niche is an essential microenvironment which evolves and responds to the physiological demands of HSCs. It is responsible for orchestrating the fate of HSCs and tightly regulates the processes that occur in the BM, including self-renewal, quiescence, engraftment, and lineage differentiation. However, the BM HSC niche is disturbed following hematological stress such as hematological malignancies, ionizing radiation, and chemotherapy, causing the cellular composition to alter and remodeling to occur. Consequently, hematopoietic recovery has been the focus of many recent studies and elucidating these mechanisms has great biological and clinical relevance, namely to exploit these mechanisms as a therapeutic treatment for hematopoietic malignancies and improve regeneration following BM injury. The sympathetic nervous system innervates the BM niche and regulates the migration of HSCs in and out of the BM under steady state. However, recent studies have investigated how sympathetic innervation and signaling are dysregulated under stress and the subsequent effect they have on hematopoiesis. Here, we provide an overview of distinct BM niches and how they contribute to HSC regulatory processes with a particular focus on neuronal regulation of HSCs under steady state and stress hematopoiesis.

## Structural components of the bone marrow niche

The bone marrow (BM) is highly vascularized to provide nutrients and oxygen. The nutrient artery and vein infiltrate the compact bone and subsequently branch to form small arterioles. These arterioles connect via transition zone vessels (also called type H capillaries) to the venous sinusoids near the endosteum, which is the interface between the bone surface and the BM ^1– 3^. BM sinusoids form a complex network and are found in the central marrow, operating as the site where hematopoietic cells migrate in and out of the BM ^4^, although a recent study suggested that transcortical vessels in bone represent an additional important migration route ^5^.

Each niche likely operates different functions and exhibits its own cellular composition. Key cellular niche components are the stromal cells necessary for producing niche factors that directly act on hematopoietic stem cells (HSCs). Perivascular cells identified by the expression of the intermediate filament protein NESTIN contain BM mesenchymal stem cells (MSCs) and can be divided in *Nestin-Gfp* transgenic mice into two subsets according to their GFP expression: Nes-GFP ^bright^ and Nes-GFP ^dim^. Nes-GFP ^dim^ cells are located around the sinusoids, and Nes-GFP ^bright^ cells are located around the arterioles ^2^ and the transition zone vessels ^6^. Stromal cells can be further divided to neuron-glial antigen (NG2)-expressing cells ^2^, Cxcl12-abundant reticular (CAR) cells ^7^, and cells expressing leptin receptor (LepR) ^1^, all of which overlap with Nes-GFP ^+^ cells to varying degrees ^8, 9^.

NG2 ^+^ cells ensheath the arterioles which have been proposed as an important niche for regulating the quiescence of HSCs via the secretion of Cxcl12, whereas others have attributed the same function to LEPR ^+^ cells in the sinusoids ^2, 10– 12^. Most likely, the discrepancies are due to different interpretations of the specificity and recombination efficiency of the Cre lines used, given the large overlap among these cell populations ^8, 9^. On the other hand, CAR cells are defined by Cxcl12 expression, essentially coincide with LEPR ^+^ cells and Nes-GFP ^dim^ cells, and are located throughout the BM ^1, 7^.

The BM is highly innervated by various types of nerves, of which the autonomic branch is predominant ^13^. Sympathetic nerve fibers enter the BM through the nutrient foramen and are closely associated with the blood vessels, before sprouting and innervating different BM regions ^14^, although some nerves may reach the BM associated with transcortical vessels in bone. The sympathetic nervous system (SNS) has been demonstrated to regulate various hematopoietic cell functions directly or indirectly mainly via the stromal cells, mediated by neurotransmitters binding to beta adrenergic receptors (β-ADRs) ^13^. β-ADRs are coupled with G _s_ trimeric proteins and activate adenylate cyclase, catalyzing the formation of cyclic adenosine monophosphate, which subsequently activates protein kinase A phosphorylation of the receptor ^15^. Contrastingly, the presence of the parasympathetic nervous system (PNS), another branch of the SNS, within the BM is vastly unexplored. The PNS uses acetylcholine (ACh) as the main neurotransmitter, which binds to muscarinic or nicotinic receptors. One study suggested that the PNS may innervate the distal femoral metaphysis ^16^ and another similarly supported cholinergic innervation within the BM of rats ^17^. However, additional neuroanatomical evidence of parasympathetic BM innervation is essentially lacking ^18^. Moreover, the bone anabolic effect of the PNS ^16^ was suggested by another group to be indirectly mediated through the inhibition of central sympathetic tone ^19^. Therefore, clarification on whether the PNS innervates the BM is required. Overall, little is known about how parasympathetic or, more broadly, cholinergic signaling might influence either HSCs or their BM niches.

## Bone marrow hematopoietic stem cell niche: location matters

The dissection of BM niches is still a developing area because of the dynamic features of the niches to meet the physiological demands and their alterations in different scenarios such as aging, malignancies, or response to stress. Single-cell studies have provided insights into the heterogeneity of the stromal cells, forming an increasingly complex picture ^20– 22^. In addition, HSCs themselves are functionally and molecularly heterogeneous ^23– 25^, raising the possibility that distinct subpopulations of HSCs are regulated by specialized niches.

It is possible that distinct vascular niches can orchestrate the balance between quiescence and proliferation of HSCs, which is necessary for homeostasis but also regeneration of the BM following injury. Consequently, studies have investigated how the regulation of HSCs differs depending on whether they are located within the endosteal region or the central marrow. In particular, these differences become more apparent under stress conditions. Following irradiation, HSCs tend to home to the endosteal region and HSCs isolated from this region exhibit greater *in vivo* homing and reconstitution potential than HSCs located in the central marrow ^26– 28^.

Furthermore, it has been demonstrated that the endosteal region is important to preserve HSC quiescence under proliferative stress and to support regeneration of the HSC pool following injury ^29– 31^. The stromal cell populations within the endosteal region better resist myeloablation, and N-cadherin ^+^ MSCs ^31^ and CD73 ^+^ MSCs ^32^ have been identified as resistant cell populations that contribute to hematopoietic stem and progenitor cell (HSPC) engraftment and subsequent hematopoietic recovery. Reserve HSCs and primed HSCs have been distinguished by their proliferation and sensitivity to chemotherapy (5-fluorouracil). Notably, whereas primed HSCs tend to be located within the central BM niche, reserve HSCs are preferentially maintained in the endosteal region ^31^. Reserve HSCs are able to resist chemotherapy in part due to N-cadherin ^+^ MSCs, which expand and produce cytokines to aid recovery after myeloablation ^31^. Overall, these studies confirm that the endosteal BM region is important for mediating hematopoietic regeneration after stress.

## Neuronal regulation under steady state

### Neuronal regulation of hematopoietic stem cells

Cumulative evidence indicates that the SNS regulates the proliferation and differentiation of HSPCs, and the migration of HSPCs and leukocytes between the BM and extramedullary sites. This was initially suggested because catecholamine levels in the blood and the BM adhered to circadian rhythms that also affected the proliferation of BM cells that expressed catecholamine receptors ^33^. More recently, Golan *et al.* demonstrated in mice that a morning peak of norepinephrine and TNF induces vascular permeability, temporarily increases reactive oxygen species (ROS) levels and facilitates HSPC proliferation, differentiation and migration. Whereas, at night, a second TNF peak increases melatonin secretion and reduces vascular permeability and HSPC ROS levels, facilitating HSPC maintenance ^34^.

A neurally-driven circadian release of HSCs and leukocytes into circulation occurs during the resting period, following photic cues ^35^. Leukocytes are also recruited to many vital organs, including skeletal muscle, following circadian oscillations of neural activity ^36^. Noradrenaline binding to β _3_-ADR on stromal cells causes a decrease in the nuclear content of Sp1 transcription factor and finally downregulation of Cxcl12 ^35^. The interaction of Cxcl12 expressed by stromal cells with its receptor Cxcr4, located on HSCs and leukocytes, is pivotal for HSC/leukocyte retention in the BM ^37^. It has also been demonstrated that the bone itself is an important transducer of signals emanating from the nervous system leading to HSC mobilization ^38, 39^.

We recently demonstrated how parasympathetic cholinergic signals coordinate with sympathetic signals to regulate the egress and homing of HSPCs and leukocytes in mice ^40^. At night, the PNS acts centrally to dampen the noradrenergic sympathetic branch and decrease BM egress of HSPCs and leukocytes mediated through β _3_-ADR ^40^. In contrast, epinephrine released at night in circulation can stimulate β _2_-ADR and increase vascular adhesion and subsequent BM homing at night ^40^. In the morning, a novel cholinergic sympathetic branch regulates vascular adhesion and β _3_-ADR expression ^40^. These results illustrate how a master rheostat SNS regulates the daily migration of HSCs and leukocytes.

## Glial cells

Glial cells supporting BM nerve fibers have also been suggested to regulate HSC proliferation ^41^. Non-myelinating Schwann cells wrap around the sympathetic nerves travelling along the vasculature within the BM. Non-myelination Schwann cells have been reported to maintain HSC quiescence via secretion of tumor growth factor beta (TGF-β) activator molecules and induction of TGF-β/SMAD signaling in HSCs ^41^. This signaling contributes to HSC quiescence through increased phosphorylation of Smad2 and Smad3 ^41^, hence supporting the maintenance and self-renewal of HSCs ^42^.

## Effects of muscarinic signaling on hematopoiesis

So far, the studies addressing cholinergic regulation of hematopoiesis have focused mostly on muscarinic receptor signaling. One study demonstrated that cholinergic receptor muscarinic 4 (CHRM4) regulated self-renewal of early erythroid progenitors, and muscarinic receptor antagonists have been proposed as a therapy for treating anemia ^43^. Pierce *et al*. uncovered another pathway connecting the brain with the BM to regulate mobilization of HSCs enforced by granulocyte colony-stimulating factor (G-CSF) ^44^. The authors demonstrated how the muscarinic receptor type 1 (Chrm1) signaling in the hypothalamus promoted G-CSF-induced HSC mobilization via the hypothalamic-pituitary-adrenal (HPA) axis ^44^. Thus, priming HSC migration through glucocorticoid (GC) hormone levels, which exhibit circadian oscillations, and binding to the receptor Nr3c1 regulates G-CSF-induced HSC mobilization ^44^. Whereas these studies have uncovered the influence of muscarinic signaling on HSCs, further investigation of other cholinergic signaling pathways (particularly involving nicotinic receptors) possibly influencing HSCs is warranted.

## Neuronal hematopoietic stem cell regulation under stress

Hematological stress can be caused as a result of a diverse range of factors from psychological stress to hematological malignancies. However, one common consequence is the dysregulation of the SNS, predominantly affecting myelopoiesis. The following sections discuss current studies of neuronal regulation of HSCs under various stress conditions.

## Immunity

Leukocytes exit the blood following circadian rhythms and undergo extensive interactions with endothelial cells as they migrate between the BM and extramedullary sites. The expression of adhesion molecules, chemokines and their receptors follow daily rhythms that regulate the migration of leukocyte subsets within distinct vascular beds. Ablation of the transcription factor BMAL1, which is an essential for clock gene, demonstrated that rhythmic leukocyte recruitment is dependent on both cell-autonomous and microenvironmental oscillations ^45^. Under stress conditions (e.g. jetlag or transplantation), alterations in these rhythms can have physiological consequences by disrupting hematopoietic cell recruitment and recovery. Therefore, time-based therapeutics for transplantations and inflammatory diseases may prove beneficial ^36^. In addition, leukocyte adhesion to arteries and veins is disproportionately disrupted following an inflammatory response, with arteries driving rhythmic inflammatory responses within the vasculature ^46^. Altogether, these studies suggest an important influence of circadian rhythms in immune response.

Mak and Tracey’s laboratories have pioneered research into how neural signals regulate immunity by showing that norepinephrine-induced T cell-derived ACh regulates immune response ^47– 50^. Recently, they have demonstrated that ChAT is induced in both CD4 ^+^ and CD8 ^+^ T cells during infection in an interleukin-21 (IL-21)-dependent manner and is key for overcoming infections ^47^. Moreover, they have validated that ChAT is expressed and ACh is produced by B cells following stimulation with sulphated cholecystokinin, resulting in controlled recruitment of neutrophils ^48^. In the proposed circuit, the vagus nerve acts via the splenic nerve, which releases ACh from T cells. ACh binds to the nicotinic ACh receptor α7 subunit on macrophages, causing the inhibition of tumor necrosis factor release, thus regulating inflammation ^49^. The vagus nerve-to-spleen circuit can also be controlled at a central level and can be exploited to suppress pro-inflammatory cytokine release ^51, 52^. Overall, these studies suggest the importance of the cholinergic vagus nerve-to-spleen anti-inflammatory pathways. However, it is important to mention that other groups have suggested alternative ways to explain the cholinergic anti-inflammatory reflex. Particularly, the efferent arm of the inflammatory reflex seems to involve a different splanchnic anti-inflammatory pathway ^53^.

## Impact of the autonomic nervous system on the skeleton

Apart from the direct regulation of hematopoietic cells and their niches, it is likely that circadian oscillation of neural activity indirectly regulates hematopoiesis and immunity through their effects on bone remodeling. Skeletal remodeling comprises two phases: resorption by osteoclasts and formation by osteoblasts, allowing vertebrates to regulate bone mass daily. Osteoblasts are multifunctional cells able to control osteoclast differentiation. Notably, the energy expenditure hormone leptin inhibits bone formation through a neuronal relay. Sympathetic signaling via β _2_-ADR on osteoblasts regulate their proliferation and control bone formation downstream of leptin ^54^. The SNS favors bone resorption by increasing the expression of Rankl on osteoblast progenitor cells, which regulates osteoclast differentiation. Moreover, leptin regulates the expression of the neuropeptide cocaine amphetamine regulated transcript (CART), which inhibits bone resorption by controlling Rankl expression ^55^.

In addition, osteoclast function is inhibited by cholinergic parasympathetic signals that inhibit sympathetic tone centrally ^19^. The skeleton can in turn modulate neural activity through secretion of the hormone osteocalcin, which regulates parasympathetic tone ^56^. Therefore, it is likely there is an interplay between sympathetic and parasympathetic nervous systems to regulate bone remodeling and stress responses. These pathways, which could have potential therapeutic implications for several complex disorders including osteoporosis, chronic fatigue and fracture repair ^57^, may also profoundly impact hematopoiesis.

## Cardiovascular disease

Ischemic myocardium causes the heart to initiate the influx of circulating myeloid cells to the site of damage. In turn, this results in the SNS signaling to the BM to increase the production of leukocytes to meet the demand, aided by circulating mediators such as granulocyte-macrophage colony-stimulating factor (GM-CSF) and IL-1β produced by the heart ^58– 61^. Therefore, the SNS regulates inflammation in cardiovascular disease by controlling HSPC proliferation and differentiation in response to stress. In addition, it was recently demonstrated that patients who exhibit recurrent myocardial infarction have a dampened emergency hematopoiesis response, due to long-term reprogramming of myeloid progenitors from the first myocardial infarction, resulting in fewer leukocytes being recruited to the site of injury ^62^. Consequently, this is a potentially important factor to consider when selecting therapies for recurrent myocardial infarction ^62^.

## Social and psychological stress

Both social and chronic psychological stress have been demonstrated to lead to SNS-induced upregulation of myelopoiesis in mice and subsequently to increases in the production of pro-inflammatory cytokines ^63, 64^. These effects were reversed with the treatment of propranolol, which is a non-selective beta blocker ^63, 64^. Following on from those studies and previous studies on adrenergic HSC regulation ^35^, chronic psychosocial stress was demonstrated to act on the most primitive progenitors, causing an increase in the proliferation of HSPCs in the BM of mice ^65^. This finding translated to the human setting, where it was observed that chronic stress induced monocytosis and neutrophilia in humans ^65^. This was due to the activation of the SNS, causing an increase in catecholamine levels, which activate the β _3_-ADR on BM niche cells, resulting in a decrease in CXCL12 levels ^35^. The HSC mobilization to peripheral circulation and the spleen, and the subsequent myeloid expansion in the spleen can aggravate chronic inflammatory diseases such as atherosclerosis ^58, 66^.

## Burn injury

In the context of burn patients, who receive multiple blood transfusions for the treatment of anemia, increased catecholamine levels induced expansion of HSPCs and increased their myeloid regulatory transcription factor (MafB) expression, causing a myeloid shift at the expense of megakaryocyte-erythrocyte progenitors ^67^. Chronic propranolol treatment restored the expansion of these cells but also influenced the myelo-erythroid bifurcation by reducing the granulocyte-monocyte progenitors and increasing megakaryocyte-erythroid progenitor cells in the BM of burn-stressed mice ^67^. These observations translated to the human setting, where *ex vivo* culture of burn patient peripheral blood mononuclear cells also demonstrated that their commitment stage of erythropoiesis was impaired and could be restored with propranolol ^67^. Consequently, beta-adrenergic blockers exhibit therapeutic value for burn patients by redirecting the hematopoietic commitment toward erythroid lineage via decreased MafB expression in multipotent progenitors, leading to increased erythropoietin responsiveness ^67^. How these forms of stress affect hematopoiesis through the nervous system is summarized in Table 1.

**Table 1.** Short-term/temporary stress affects hematopoiesis through the nervous system.

Form of stress	Experiment	Effect on neuronal regulation	Effect on hematopoietic system	Blocked by	References
Social stress	Human: Analyzed peripheral blood mononuclear cells from patients from high versus low socioeconomic status	↑ Catecholamines ↑ β-adrenergic transcription factor cAMP	↑ Pro-inflammatory genes (e.g., interleukin 1 beta [IL- 1B], tumor necrosis factor, and IL-8) ↑ Monocytes		63
	Mice: Six daily cycles of 2-hour exposure to an aggressive male intruder mouse		↑ Granulocyte-macrophage colony-stimulating factor receptor (CSF3R) ↑ Myelopoiesis ↑ Genes involved in cell growth and differentiation	Propranolol	
Chronic psychological stress	Mice: Restrained for 5 consecutive nights during resting period without food/water	↑ Catecholamines	Severe leukocytopenia and immunosuppression	Propranolol	64
Psychosocial stress	Human: Blood samples taken from medical residents after 10 consecutive days off duty versus 7 consecutive days on duty		Higher number of leukocytes		65
	Mice: Extended cage tilt, placing in small individually cages prior to moving to overcrowded cage, damp bedding, light-dark changes, overnight illumination	↑ Noradrenaline levels ↓ Bone marrow (BM) CXCL12 mRNA and protein	↑ Leukocyte production ↑ Cycling of hematopoietic stem cells (LSK CD150 ^+^CD48 ^−^) ↑ BrdU incorporation ↓ BrdU label retention ↑ Colony-forming capacity	β _3_-ADR knockout mice or treatment with β _3_- selective receptor blocker	
	Mice: 5-fluorouracil (5-FU) challenge: 3-week stressed versus non-stressed		↑ Leukocyte rebound on day 14 after 5-FU injection		
Burn injury	Human: Burn patients	↑ Catecholamines	↑ Anemia ↓ Megakaryocyte-erythroid progenitor cells (MEPs)	Propranolol	67
	Mice: Anesthetized mice were subjected to a 15% total burn surface area scald burn by immersion in a 100° water bath for 9 seconds.	↑ Catecholamines	↑ LSK numbers ↑ Myelopoiesis ↓ MEPs	Propranolol for 6 days	

LSK, Lin-Sca1 ^+^ckit ^+^.

## Aging

The hematopoietic system is disrupted upon aging, resulting in the increase of HSCs which are functionally impaired ^68, 69^. These changes are caused by both cell-intrinsic dysregulation and remodeling of the BM microenvironment ^70, 71^. One of the hallmarks of hematopoietic aging is that the myeloid output of HSCs increases at the expense of lymphopoiesis ^68, 69^. One study carried out transplantations of old HSCs into young recipients and vice versa. The young microenvironment was able to reduce myelopoiesis, confirming the contribution of the microenvironment ^72^. We recently noted that the SNS is actively involved in aging of the hematopoietic system. Our recent study demonstrated that sympathetic noradrenergic fibers marked by tyrosine hydroxylase (Th) increased in the mouse BM with aging ^6^. Increased β _2_-adrenergic signaling in expanded central BM niches promoted myeloid cell expansion ^6^. A functional switch of neurotransmission, favoring β _2_-ADR over β _3_-ADR signaling during aging, appears to favor myeloid cell expansion through the regulation of the BM microenvironment ^6^. An active role for the nervous system in aging is supported by the increased basal sympathetic tone during human aging ^73– 75^ and by a recent study indicating that increased excitatory neurotransmission reduces the life span ^76^. In contrast, another study suggested that BM Th ^+^ fibers were reduced (not increased) during aging and that surgical denervation of young BM increased myelopoiesis ^77^. However, BM noradrenergic nerve fibers appear to decrease from youth to adulthood (8-month-old adult mice compared with 2-month-old mice; Supplementary Figure 5b in REF ^77^) but these fibers appear increased (not decreased) in old (20-month-old) mice ^6^. Moreover, the possible contribution of the inflammation caused by experimental surgical denervation to the hematopoietic aging phenotypes ^77^ should be considered.

## Diabetes

The disautonomia associated with diabetes has been shown to affect the BM, disrupt the peripheral clock, and compromise G-CSF-induced HSC mobilization in experimental models because of the HSC niche deregulation ^78, 79^. In humans, cardiovascular diabetic autonomic neuropathy correlates with decreased circulating HSPCs with increased 66-kDa protein from the src homology and collagen homology domain (p66Shc) and reduced expression of sirtuin 1 (Sirt1) ^80^.

## Hematopoietic recovery following radiation

Ionizing radiation and chemotherapy used to treat cancer cause BM injury and alter the BM cellular composition. Following chemotherapy, there is an increase in apoptosis of mature cells along with progenitor cells that are cycling. Chemotherapy can damage BM innervation ^81^ and catecholamines, namely norepinephrine can improve hematopoietic reconstitution following chemotherapy in mice ^81, 82^. Furthermore, G-CSF and GM-CSF are commonly used to accelerate myelopoiesis and minimize the burden of chemotherapy. These cytokines upregulate the expression of neuronal receptors on HSPCs, allowing for them to form a greater response to neurotransmitters, leading to enhanced proliferation and motility of human CD34 ^+^ progenitor cells and subsequent repopulation of mouse BM ^83^. Additionally, mitotically active Nestin-GFP ^+^ perisinusoidal niche cells are greatly diminished whereas the Nestin-GFP ^+^ peri-arteriolar niche cells exhibit greater chemoresistance because of their higher quiescence ^2^.

Consequently, the use of adrenergic agents as a therapeutic approach should be investigated further, adding to the available evidence on α1-ADR agonists or β-ADR agonists ^81, 82, 84^.

## Hematological malignancies

**Figure 1.**
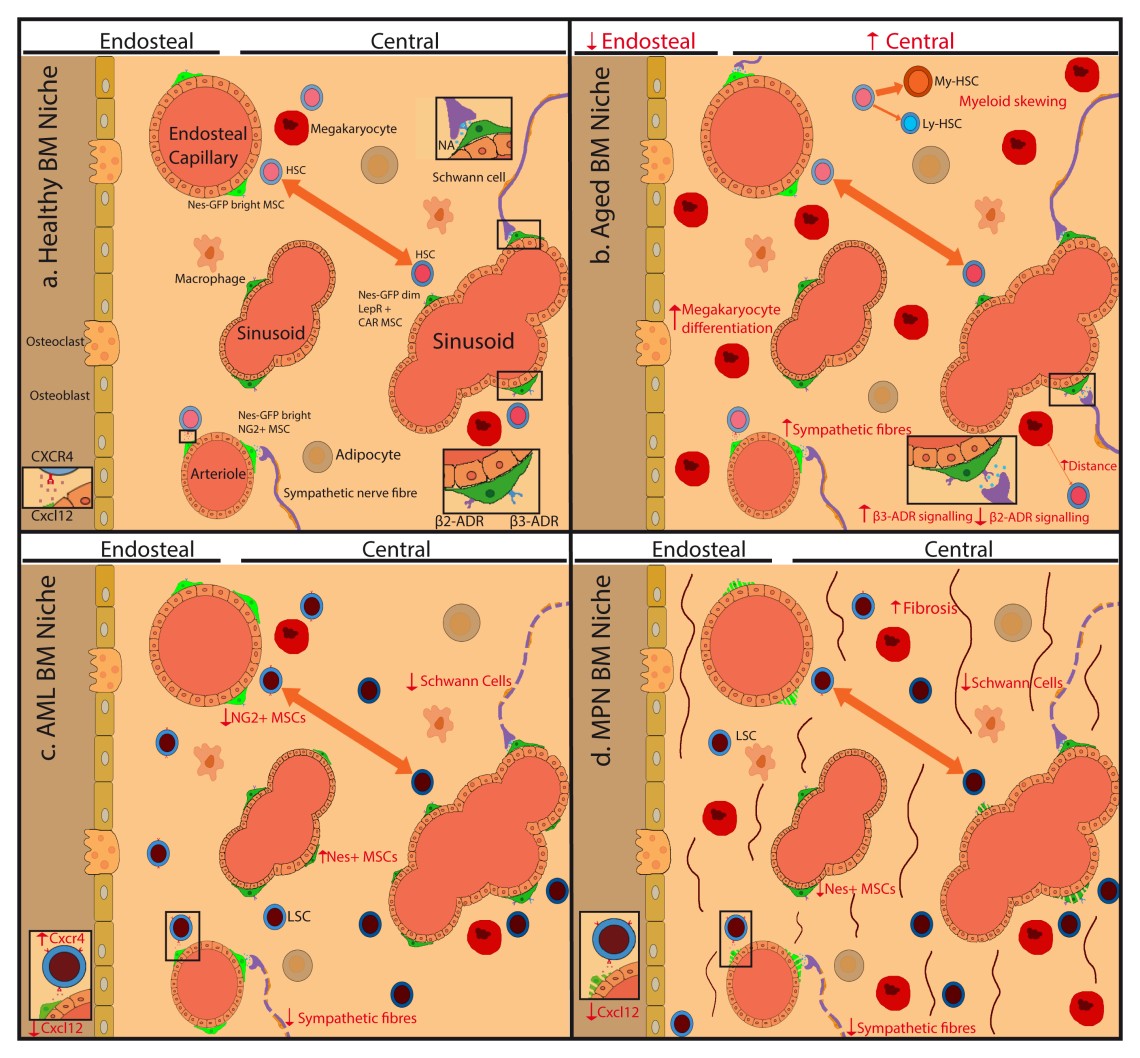
Model illustrating hematopoietic stem cell niche alterations with aging and age-related myeloid malignancies. Scheme shows key hematopoietic stem cell (HSC) niche cell types and their alterations during aging and age-related myeloid malignancies. ( **a**) Mesenchymal stem cells (MSCs), which can differentiate into osteoblasts or adipocytes, regulate HSCs in endosteal niches enriched in transition zone capillaries or in central niches enriched in sinusoids. Nestin-green fluorescent protein (Nes-GFP) ^bright^ neural-glial antigen 2 (NG2) ^+^ MSCs are associated with endosteal capillaries and arterioles located throughout the bone marrow (BM), whereas Nes-GFP ^dim^ leptin receptor (LEPR) ^+^ CXC-chemokine ligand 12 (CXCL12)-abundant reticular (CAR) MSCs are associated with sinusoids in the central BM. Sympathetic nerve fibers regulate CXCL12 expression in MSCs and the migration of HSCs through the sinusoids. Different MSC subpopulations, endothelial cells, non-myelinating Schwann cells, and megakaryocytes contribute to regulate HSC proliferation. ( **b**) During mouse aging, sympathetic fibers increase, but β _3_-adrenergic signaling is reduced, whilst β _2_-adrenergic signaling increases promoting myeloid skewing. Megakaryocytes increase but locate further away from HSCs. ( **c**, **d**) In myeloid malignancies, a damage to this neural regulation of MSCs might contribute to disease progression. (c) In acute myeloid leukemia (AML), sympathetic nerve fibers and NG2 ^+^ Nes-GFP ^bright^ MSCs decrease, whilst Nes-GFP ^dim^ MSCs increase, although the implications for human AML are unknown. (d) In MPN, the neuroglial damage leads to apoptosis of Nestin-GFP ^+^ MSCs, which can be rescued through chronic treatment with sympathicomimetic drugs that indirectly improve reticulin fibrosis in mice and humans. ADR, adrenergic receptor, NA, noradrenaline.

The SNS has also been implicated in the development of hematological malignancies, predominantly the progression of myeloid malignancies. Sympathetic neuropathy occurs in the development of both acute myelogenous leukemia (AML) and myeloproliferative neoplasms (MPNs) ^85, 86^, but the consequences appear to be different. In MPN, IL-1β produced by the mutant hematopoietic cells damages sensitive HSC niche components, such as neural terminals, Schwann cells, and Nestin ^+^ MSCs ^86^. In contrast, experimental AML causes the reduction in arteriole-associated NG2 ^+^ cell numbers and correlates with the expansion of Nestin-GFP ^+^ stromal cells ^85^. In MPN, chronic administration of β _3_-adrenergic agonists to compensate for the defective innervation can rescue Nestin ^+^ niche cells and improve myelofibrosis (BM scarring hampering normal hematopoiesis) in both mice ^86^ and humans ^87^. Different effects of β _3_-adrenergic agonists in mouse models and human MPN might be explained by the different drugs or dosing used. In AML, the relevance of sympathetic neuropathy ^85^ remains to be demonstrated in humans. The changes in sympathetic regulation of HSC niches during aging and age-related myeloid malignancies are briefly summarized in Figure 1.

## Conclusions and future perspectives

The BM is regulated by neural signals principally emerging from the autonomic nervous system. The sympathetic noradrenergic branch has been much more explored than the parasympathetic (cholinergic) branch, both under steady state and during stress hematopoiesis. The data available suggest that sympathetic innervation regulates BM homeostasis but is especially important to respond to stress scenarios. Recent evidence suggests that the cholinergic branch of the autonomic nervous system contributes to this regulation ^40^. However, the roles of this cholinergic branch (sympathetic or parasympathetic) in the regulation of hematopoiesis remain largely unexplored.

During chronic inflammation, cardiovascular disease, and short-term social and psychological stress, beta blockers have been demonstrated to revert excessive myelopoiesis. Whether a similar strategy could be proposed to prevent excessive myeloid cell production during aging or age-related myeloid malignancies requires further investigation. The possible contribution of other adrenergic or cholinergic signaling pathways to the progression of hematological disorders is an exciting area for future investigation.

## Abbreviations

ACh, acetylcholine; ADR, adrenergic receptor; AML, acute myelogenous leukemia; BM, bone marrow; CAR, Cxcl12-abundant reticular; ChAT, choline acetyltransferase; G-CSF, granulocyte colony-stimulating factor; HSC, hematopoietic stem cell; HSPC, hematopoietic stem and progenitor cell; IL, interleukin; LepR, leptin receptor; MafB, myeloid regulatory transcription factor; MPN, myeloproliferative neoplasm; MSC, mesenchymal stem cell; NG2, neuron-glial antigen; PNS, parasympathetic nervous system; SNS, sympathetic nervous system; Th, tyrosine hydroxylase
